# Characterization, Shear Bond Strength Assessment, and Antibacterial Effects of an Orthodontic Composite Containing Stannous Oxide (SnO2) Nanoparticles

**DOI:** 10.7759/cureus.54977

**Published:** 2024-02-26

**Authors:** Niharika Bhatia, Navaneethan R, Pugalmani S

**Affiliations:** 1 Orthodontics and Dentofacial Orthopedics, Saveetha Dental College and Hospitals, Saveetha Institute of Medical and Technical Sciences, Saveetha University, Chennai, IND

**Keywords:** antibacterial property, shear bond strength, composites, orthodontic adhesive, nanoparticles

## Abstract

Background

This study aimed to determine the antibacterial properties of orthodontic adhesive infused with stannous oxide nanoparticles (NPs) against *Lactobacillus acidophilus* and *Streptococcus mutans* bacteria, along with assessing the shear bond strength (SBS) of this composite when compared to conventional, non-infused composites.

Methods

A concentration of 1% w/w tin dioxide NPs (SnO_2_ NPs) was added to Transbond XT Orthodontic Adhesive. This modified composite material was used to prepare composite discs for the evaluation of its antibacterial properties against *L. acidophilus* bacteria and *S. mutans* bacteria using the biofilm inhibition test. To evaluate the SBS of this modified adhesive material, 50 extracted premolar teeth were collected and divided into two groups, with 25 teeth in each group (n = 25). Orthodontic stainless steel brackets were bonded to these extracted teeth using the modified composite. A comparative analysis of the SBS of the nano-infused composite group was then performed against that of the control group using an Instron universal testing machine.

Results

Growth inhibition zones were produced around the composite discs infused with SnO_2_ NPs for both bacterial strains. After performing the biofilm inhibition test, it can be inferred that the nano-infused composite is capable of inhibiting the bacterial count better than the control group. A statistically significant difference was observed between the two groups, with the SBS of the nano-infused composite being higher (16.89 MPa) than the non-infused composite adhesive (15.49 MPa).

Conclusion

The antibacterial activity of orthodontic composites modified with SnO_2_ NPs was significant compared with conventional composites. The control group showed less SBS when compared to the NP-infused composite, with a statistically significant difference in mean SBS values between both groups.

## Introduction

In the field of orthodontics, advancements in material science have played a pivotal role in revolutionizing treatment modalities and enhancing patient outcomes. The use of orthodontic composites in bonding brackets and other appliances has become a cornerstone of contemporary orthodontic practice, offering superior aesthetics, improved bond strength, and reduced chairside time. However, a common problem faced by individuals undergoing orthodontic treatment was the occurrence of demineralization of the enamel surface throughout fixed braces therapy [1]. Plaque accumulation is facilitated by brackets and fixed orthodontic attachments as they provide retentive regions [2]. Rapid changes in the microbiota of plaque brought on by *Streptococcus mutans*, *Staphylococcus aureus*, and *Lactobacillus* result in increased amounts of an acidogenic environment [3], which causes the demineralization of the enamel and the development of subsequent white spot lesions [4]. The formation of lesions can occur as early as four weeks, emphasizing the significance of preventing and managing them [5]. The incorporation of advanced materials into orthodontic procedures has experienced substantial progress in the past few years [6]. Recently, in the dental and medical sectors, there has been a lot of interest in using particular nanoparticles (NPs) as antibacterial agents [7]. These NPs possess a unique physicochemical composition, which enables them to interact more closely with the surfaces of bacterial cells, which are known to be negatively charged, thereby enhancing their antibacterial activity. To further optimize the performance of orthodontic composites, researchers have explored the incorporation of nanomaterials, such as NPs, to impart unique properties and functionalities [8]. Prior research conducted by Ahn et al. [9] has demonstrated that the incorporation of silver nanofillers in orthodontic adhesives can effectively inhibit enamel demineralization while maintaining the material’s physical characteristics intact. The ability of nanocomposites and nanoionomers to meet the shear bond strength (SBS) levels regarded as clinically acceptable in previous recommendations makes them viable for bonding applications [10]. The use of these NPs has been shown to have a significant therapeutic impact on* S. mutans*, which is well known for its sensitivity toward zinc oxide, silver, gold, and titanium [11]. Tin dioxide NPs (SnO_2_ NPs), one of the newest nanomaterials, have drawn a lot of interest because of their excellent physicochemical characteristics and potential uses in a variety of biomedical sectors. SnO_2_ NPs possess a large surface-to-volume ratio, a high surface area, and potent antimicrobial properties, making them a compelling candidate for use in orthodontic composites. Hence, this investigation aimed to evaluate and compare the antibacterial properties and SBS of the SnO_2_-infused nanocomposite with the traditional, non-nano-infused composite. This study has been performed with the primary objective of evaluating the SBS of the nano-infused composite and a secondary objective of assessing the antibacterial activity of the same.

## Materials and methods

**Figure 1:**
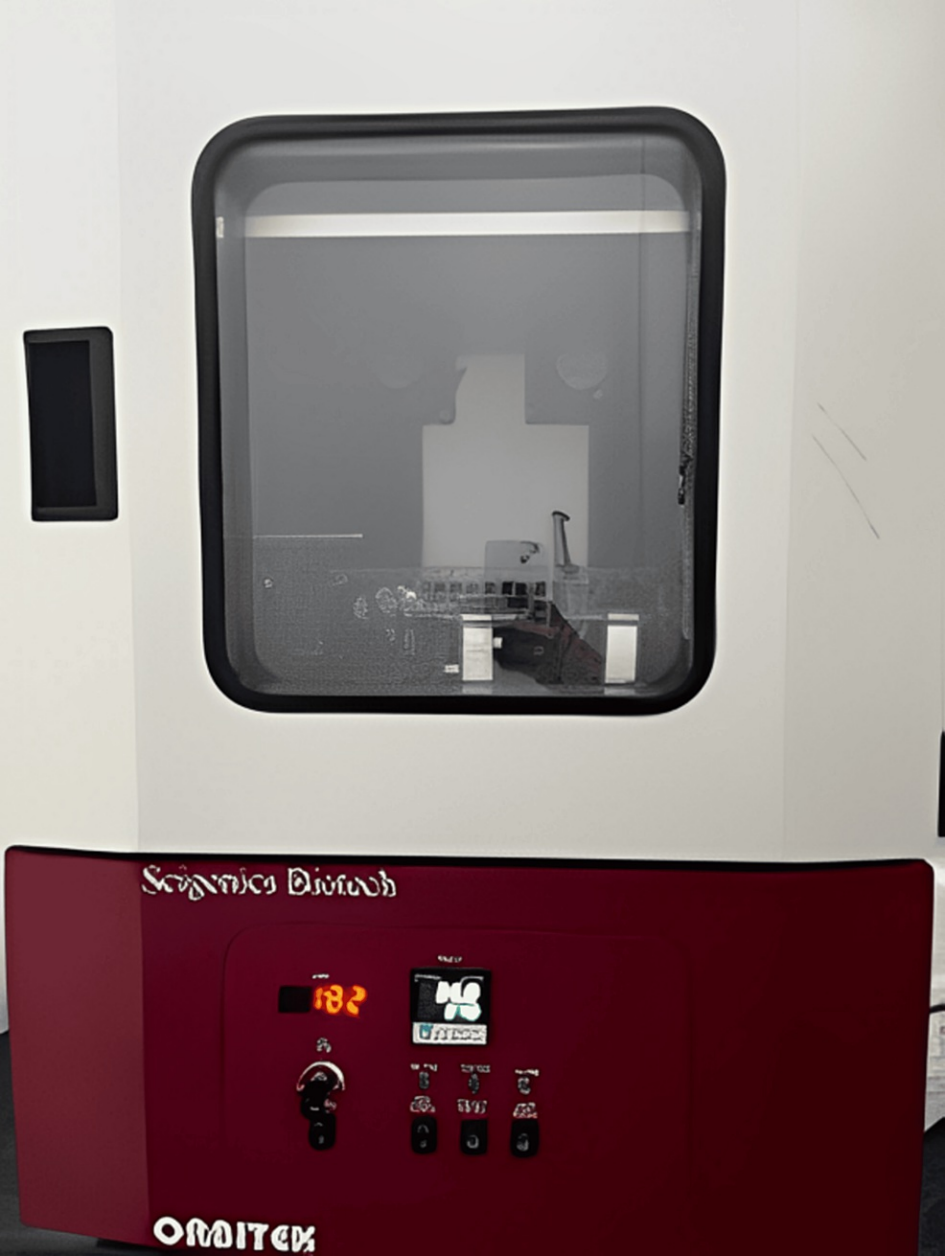
Ball Mill Composite Mixer used in this study

This study was conducted at Saveetha Dental College and Hospitals, Chennai, India. Commercially available SnO_2_ NPs (dry nanopowder with an average primary particle size of 30-50 nm and purity >99.5%) were mixed into an orthodontic composite (3M™ Transbond™ XT Light Cure Adhesive, 3M Company, Saint Paul, Minnesota, United States) at an optimal weight (%) in a dark environment using a High Energy Ball Mill Composite Mixer (Retsch Emax, Retsch GmbH, Haan, Nordrhein-Westfalen, Germany) (Figure 1).

**Figure 2:**
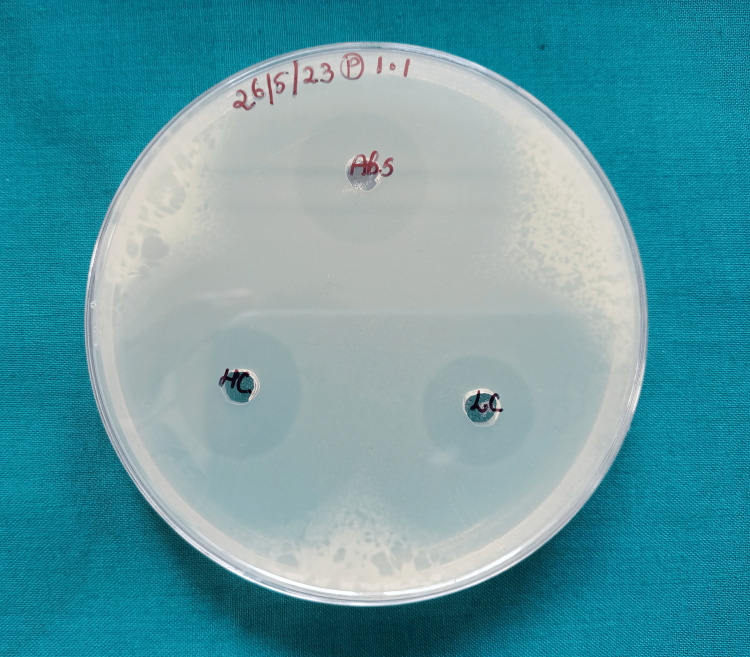
The agar plate showing zones of inhibition Abs, LC, and HC marked on the agar plate are standard antibiotics used for antimicrobial testing.

For the current study, 50 composite discs were prepared for testing. The sample size of 50 was based on the study by Reddy et al. [12] performed using the G*Power Software Version 3.1 with a power of 95%. Each of the 50 composite disks used in the study was poured into a mold with a thickness of 3 mm and a diameter of 6 mm to create discs of equal dimensions. A clear thermoplastic sheet was used to take these measurements (Bioplast Manufacturing, L.L.C., Bristol, Pennsylvania, United States). *S. mutans *(MTCC 497) and *Lactobacillus acidophilus *(MTCC 10307) were tested for antibacterial properties using the disks prepared using the composite material. The disc agar diffusion test was used to evaluate the antibacterial activity. This test examines the antibacterial drugs’ capacity to permeate the agar and establish an area of bacterial inhibition. On the Mueller-Hinton agar plate, three composite discs (n = 3) were placed two centimeters apart from one another (Figure 2).

**Figure 3:**
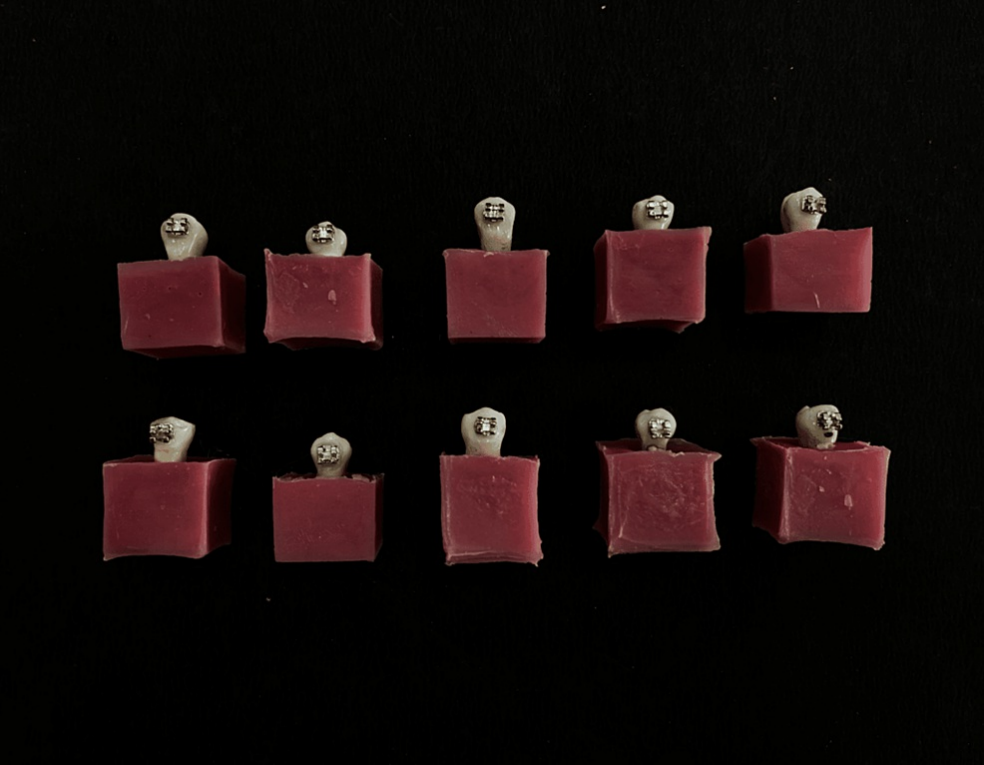
Sample preparation for evaluating SBS SBS, shear bond strength

Out of the three composite discs, one was the control, i.e., the disc made with conventional Transbond XT composite without any nanocomposite infusion, whereas the other two composite discs were of the Transbond XT composite infused with SnO_2_ nanomaterial. Next, 20 µL of bacterial suspensions (approximately 108 CFU/ml) were spread on the discs, and their density was adjusted to 0.5 on the McFarland scale using sterile phosphate buffered saline. An inhibition zone measurement scale was used to measure the width of the bacterial growth inhibition for both *S. mutans* and *L. acidophilus* after a 48-hour incubation period. A total of 30 freshly extracted premolar teeth were divided into two groups, with 15 teeth in each group. The selection of teeth was based on specific criteria, encompassing clear anatomical and morphological features of extracted premolars. These teeth needed to have an intact outer enamel surface on the buccal aspect and be devoid of any developmental irregularities, enamel decay, or fractures on the crown. Any teeth exhibiting damage such as cracks, decay, or other issues on the buccal surfaces were not included in the sample. The chosen premolar teeth were placed in PVC tubes and secured in cold cure acrylic resin. The teeth were inserted vertically into the acrylic material until they reached the cementoenamel junction, followed by the implementation of the usual bonding protocol. The buccal surface of the tooth underwent a treatment involving a 37% phosphoric acid application for 30 seconds. This was followed by a thorough 30-second rinse with flowing water and then gentle drying through an air spray. A thin layer of Transbond XT primer from 3M Unitek was applied to the enamel’s surface in both experimental groups, and the enamel was then exposed to light curing for 10 seconds using the Woodpecker DTE-O light curing unit. For the initial group, the enamel surface received Transbond XT adhesive, also from 3M Unitek, for attaching MBT 0.022-inch stainless steel premolar brackets (American Orthodontics, Sheboygan, Wisconsin, United States). Using a metal index, the brackets were precisely placed parallel to the buccal surface of the tooth. After alignment, they were cured for 20 seconds and split evenly between the distal and mesial sides of the bracket (Figure 3).

**Figure 4:**
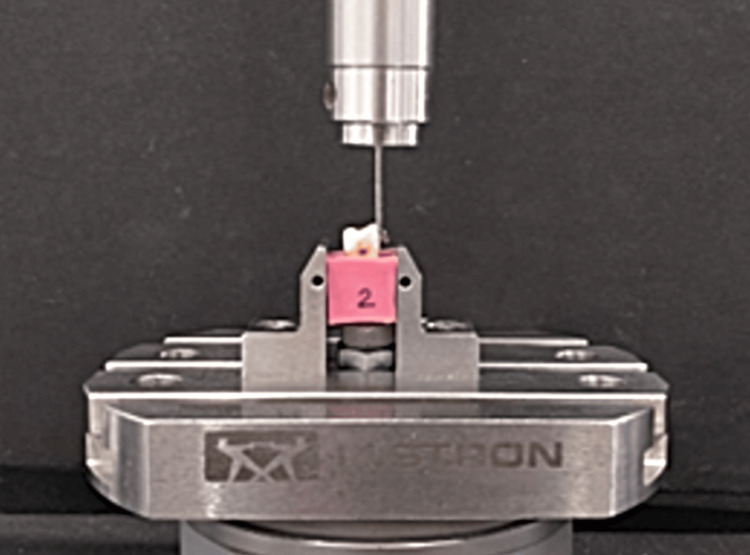
Instron universal testing machine (Instron® model 8801) equipped with a 1 kN load cell and operating at a crosshead speed of 1 mm/min was used to measure SBS SBS, shear bond strength

With the exception of using nanocomposite to facilitate the bonding process, the bonding procedure of the second group was carried out in the same manner as that of the first group. Any surplus adhesive was meticulously eliminated with the use of a probe. The teeth bonded with brackets were immersed in distilled water for 24 hours. An Instron universal testing machine (Instron® Model 8801, Instron Corporation, Norwood, Massachusetts, United States) equipped with a 1 kN load cell and operating at a crosshead speed of 1 mm/min was used to measure bond strength. An occlusal-gingival load was applied to the bracket using a blade-end steel rod linked to the machine’s crosshead. At the bracket-tooth connection, this caused a shear force to be generated. A computer connected to the Instron machine electronically recorded the moment the bracket broke under load in Newtons. The strength of the shear bond was then assessed (Figure 4).

## Results

Disc agar diffusion test

**Figure 5:**
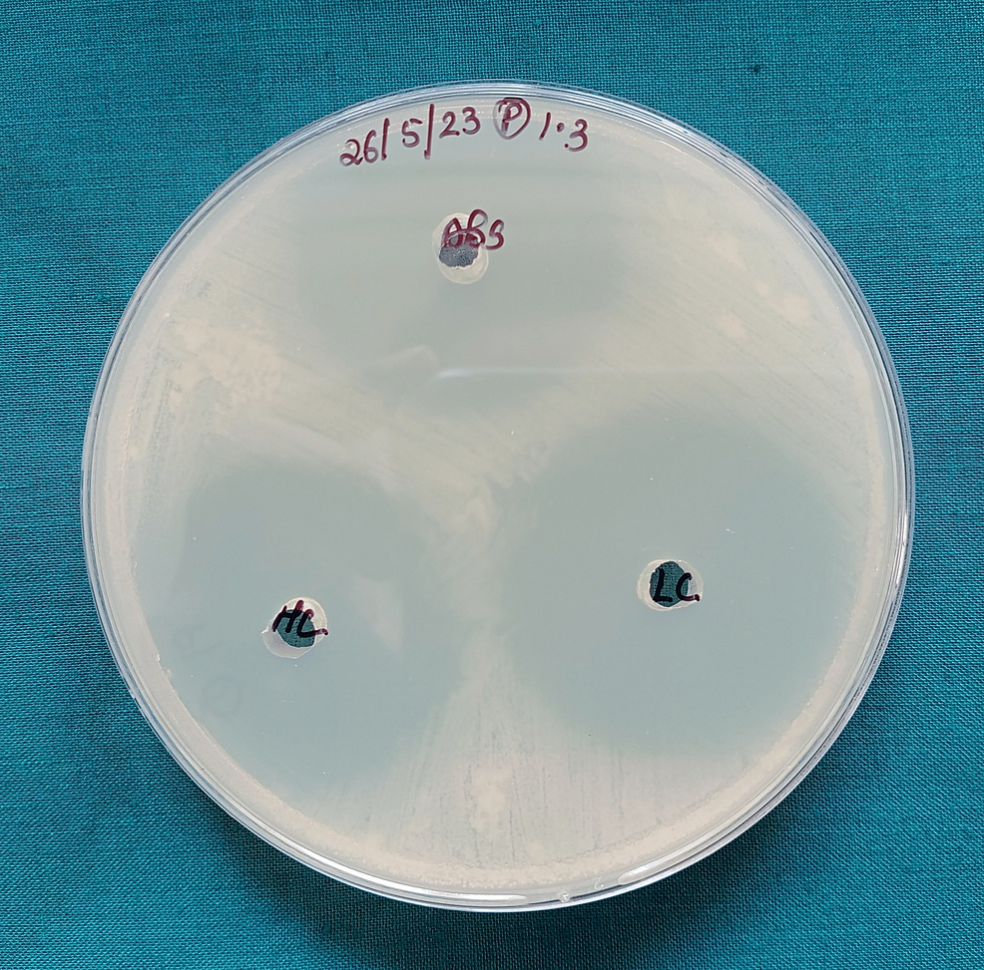
Zones of inhibition around conventional composite discs (control) The transparent circle around each composite disc is indicative of the zone with no bacterial growth. The white area is indicative of an area with bacterial growth. Abs, LC, and HC are standard antibiotics used for testing.

**Figure 6:**
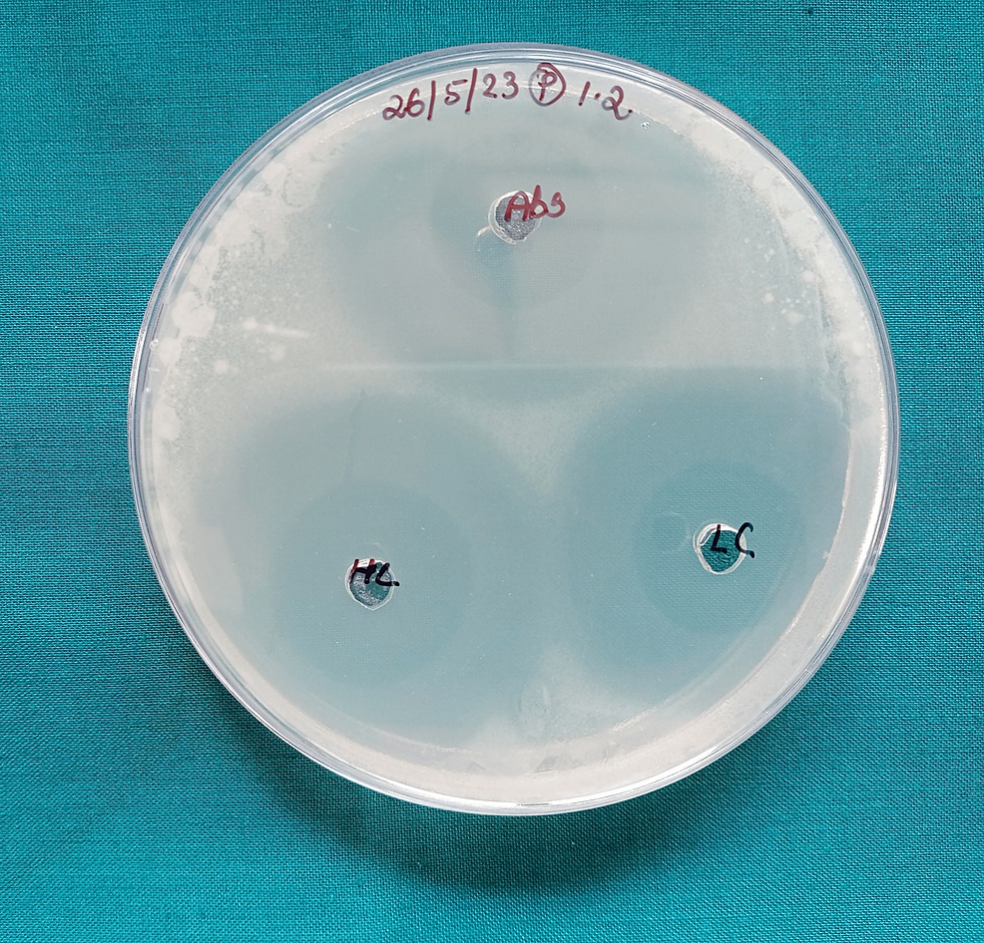
Zone of inhibition around SnO2 NPs filled composite against Lactobacillus acidophilus This figure demonstrates a higher zone of inhibition for the SnO_2_ NPs filled composite when compared to that around the control. NP, nanoparticle; SnO_2_ NP, tin dioxide NP

**Figure 7:**
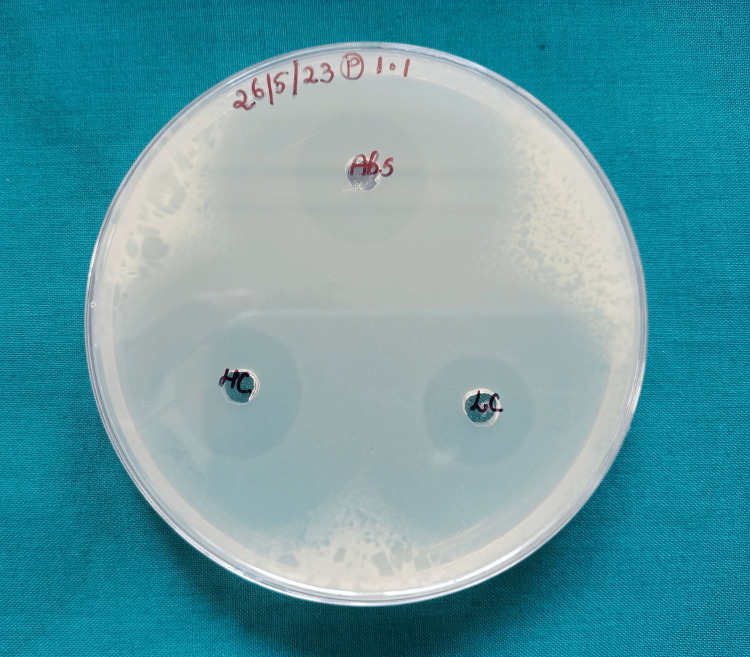
Zone of inhibition around the nanofilled composite discs against Streptococcus mutans The figure demonstrates a higher zone of inhibition around the nanofilled composite discs when compared to the control.

The disc agar diffusion test findings showed a substantial difference in the zone of inhibition against *L. acidophilus *and *S. mutans*. The zones of inhibition for both bacterial strains were notably larger around the nanofilled composite discs when compared to those around the control group composite discs, indicating a stronger antibacterial effect against these microorganisms by the nanofilled composites (Figure 5, Figure 6, and Figure 7).

SBS

SBS results were analyzed using a paired sample t-test using IBM SPSS Statistics for Windows, Version 23.0 (Released 2015; IBM Corp., Armonk, NY, USA). At a 95% confidence level, a p-value of less than 0.05 (p = 0.05) was regarded as statistically significant (Table 1 and Table 2).

**Table 1:** Descriptive statistics for the two groups (n = 15) NP, nanoparticle; SBS, shear bond strength

Groups	n (Samples per group)	Mean SBS (MPa)	SD
Group 1 (NP-infused composite)	n = 15	16.89	1.1
Group 2 (non-infused composite)	n = 15	15.49	0.68

**Table 2:** Results of paired sample t-test p < 0.05 is considered significant.

Pair	Mean	SD	Significance (p-value)
Nano-infused composite control	1.4	1.1	0

Between the two groups, there was a statistically significant difference in mean SBS (p< 0.005). When compared to the traditional (non-infused) composite group, the mean SBS of the nano-infused composite group was greater. An increase in the SBS post-infusion of the NP indicates that the orthodontic brackets will not debond easily on the application of a shear force such as biting or chewing, leading to a lesser debonding rate. An increase in the zone of inhibition, as indicated in Figure 6 and Figure 7, implies that not only will the nano-infused composite lead to a lesser bond failure rate but also provide a higher antibacterial effect against *L. acidophilus* and* S. mutans* strains, two of the most common bacteria causing white spot lesions and dental caries.

## Discussion

Composite resin adhesives continue to be the preferred option for bonding brackets among orthodontists, despite their widespread use. Although it is crucial to recognize that these adhesives can contribute to the demineralization of adjacent enamel, certain limitations persist unresolved. Notably, *S. mutans *has been identified as the primary contributor to enamel demineralization, in line with research by de Soet and de Graaff [13]. Several antibacterial agents have been incorporated into orthodontic adhesives in research to increase their antibacterial characteristics [14,15]. The rise of nanotechnology has introduced NPs as promising candidates for orthodontic bonding due to their unique attributes. As highlighted by Kim et al. [16], NPs and their ions can generate free radicals, inducing oxidative stress in the form of reactive oxygen species (ROS). These ROS can inflict irreversible damage upon bacteria, affecting their membrane, DNA, and mitochondria, ultimately leading to bacterial elimination. Several researchers have examined the impact of NP incorporation on the SBS of orthodontic adhesives. However, it is important to note that elevated NP concentrations might result in cytotoxic effects [17]. NPs are recognized for their antimicrobial effects, ascribed to their small size and substantial surface area [18]. Given their sub-100 nm size and insolubility [19], NPs possess distinct characteristics. In this study, a 1.0% w/w concentration of SnO_2_ NPs was integrated into orthodontic adhesives. The large surface area and small particle size allow for improved ion release at low filler levels without causing cytotoxicity. The selection of SnO_2_ NPs was based on their advantageous biological properties, making them attractive for various applications. These include antioxidant properties, anti-inflammatory effects, and biocompatibility. SnO_2_ NPs are known for their potent antibacterial activity against a range of pathogenic bacteria. They can hinder bacterial growth and biofilm formation, suggesting potential for addressing bacterial infections and promoting wound healing. Moreover, SnO_2 _NPs can be tailored for drug delivery applications, facilitated by their small size and biocompatibility, enhancing drug efficacy and minimizing adverse effects. In contrast to standard composites, composites treated with 1% w/w SnO_2_ NPs showed strong growth inhibition zones against *S. mutans* and *L. acidophilus*. The leached components from these modified composites exhibited notable antibacterial properties against *S. mutans* and *L. acidophilus*, resulting in a marked decrease in bacterial colony count (CFU/ml) during the initial three days. However, from day 3 to day 30, a gradual increase in colony count (CFU/ml) was observed across all three experimental groups. This marked reduction in the bacterial colony count, as indicated by the wider zone of inhibition around these nanofiller composites, indicated that this composite material is effective in resisting the growth of these two bacterial strains, thereby reducing the risk of white spot lesions and dental caries, as *L. acidophilus* and *S. mutans* are the two bacteria that most commonly lead to dental caries. This study also sought to evaluate the SBS of orthodontic adhesives containing 1% (w/w) SnO_2_ NPs and compare it with the control group. The findings indicated a significant increase in the mean SBS values of the 1% (w/w) SnO_2_ NP group compared to the conventional composite (control group). Reynolds [20] established a clinically acceptable SBS range of 6-8 MPa. However, the current investigation demonstrated that the mean SBS values of the experimental groups were above the clinically acceptable range and also above the control group (15.49 MPa), deviating from the findings reported by Reddy et al. [12]. Our findings contradicted those of Uysal et al., who found that Transbond XT had a considerably higher SBS value when comparing the SBS of a nano-composite (Filtek Supreme Plus Universal) and a nano-ionomer (Ketac ™ N100 Light-Curing Nano-Ionomer) with Transbond XT. These disparate results, however, could be the result of several things. For example, the teeth in Uysal et al.’s [10] study were polished with nanofluoridated pumice, which would have prevented nanofillers from penetrating the etched enamel surface and weakened the binding. A noteworthy distinction between their utilization of a QTH light source and our study’s employment of metal brackets and porcelain-based brackets was the latter.

There are drawbacks to this study as well. Since the study is in vitro, in vivo testing is required to reach an accurate conclusion. The Adhesive Remnant Index, which may provide information about the interface where bond breakdown is most common, is not used in the current study.

## Conclusions

The inclusion of stannous oxide NPs in adhesive substances in small quantities has the potential to influence the SBS, potentially resulting in a reduction of bracket or adhesive failure. The addition of SBS also enhances the antibacterial properties of the adhesive material, making it effective against various microorganisms, such as *S. mutans* and* L. acidophilus*.
